# Supplementary material for: Establishment of Polydopamine-Modified HK-2 Cell Membrane Chromatography and Screening of Active Components from Plantago asiatica L
Source: Int J Mol Sci. 2024 Jan 18;25(2):1153. doi: 10.3390/ijms25021153 (PMC10816010; doi:10.3390/ijms25021153)
Supplement: Supplementary file 1 [file ijms-25-01153-s001.zip › ijms-2793074-supplementary.pdf]

Establishment of Polydopamine-modified HK-2 cell membrane chromatography and screening of active components from *Plantago asiatica* L.

Hongxue Gao [a,b], Zhiqiang Liu [a,b], Fengrui Song [a], Junpeng Xing [a], Zhong Zheng [a], Zong Hou [a,*], Shu Liu [a,b,*]

[a] State Key Laboratory of Electroanalytical Chemistry & Jilin Provincial Key Laboratory of Chinese Medicine Chemistry, Changchun Institute of Applied Chemistry, Chinese Academy of Sciences, Changchun, 130022, China

[b] Institute of Applied Chemistry and Engineering, University of Science and Technology of China, Hefei, 230029, China

*Corresponding authors:

Shu Liu and Zong Hou

Changchun Institute of Applied Chemistry, Chinese Academy of Sciences, 5625 Renmin Street, Changchun 130022, Jilin, China. Tel.: +86-431-85262613; Fax: +86-431-85262886; E-mail address: liushu@ciac.ac.cn (Shu Liu); houzong@ciac.ac.cn (Zong Hou)

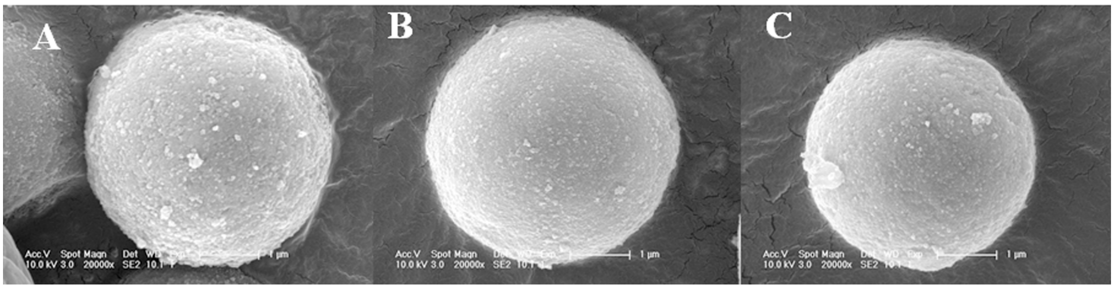

Figure S1 The SEM image. (A) silica gel, (B) silica gel after vacuum stirring, and (C) silica gel after suction filtration.

Table S1 The diameter of silica gel treated with different methods

sample	Average diameter (μm)	RSD (%)
silica gel	4.18	0.78
silica gel after vacuum stirring	3.98	0.93
silica gel after suction filtration	3.64	2.11

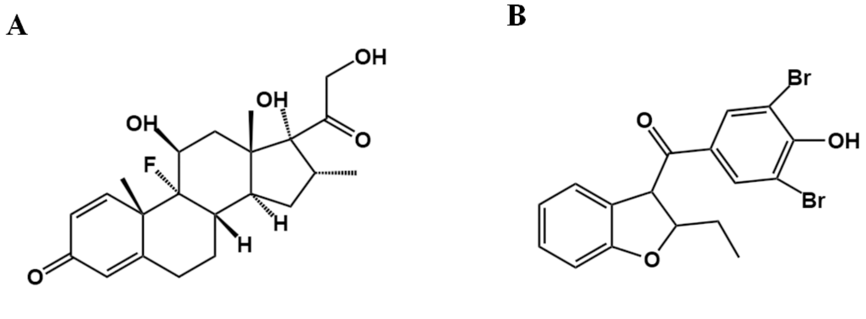

Figure S2 The chemical structure of (A) dexamethasone and (B) benzbromarone.

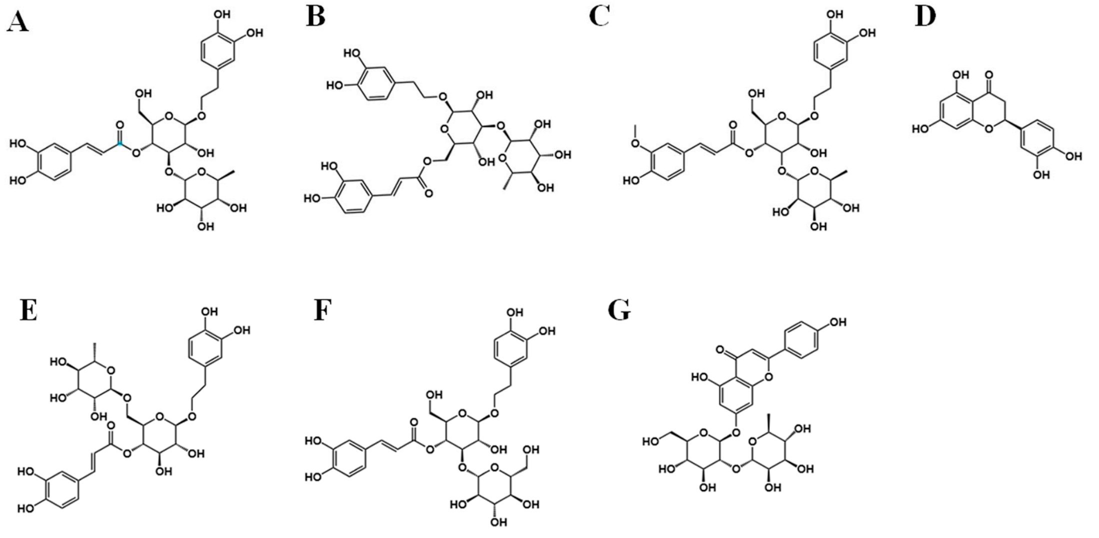

Figure S3 The chemical structure of seven potential gout treatment components selected from *Plantago asiatica* L. (A) acteoside, (B) isoacteoside, (C) leucoseptoside A, (D) eriodictyol, (E) forsythiaside A, (F) plantamajoside, (G) rhoifolin.

Table S2. The precursor ions, product ions, and voltage parameters

**Analytes**	**Precursor *m/z***	**Product *m/z***	**Q1(V)**	**Q3(V)**
Uric acid	167.10	124.15	19	12
Hippuric acid	178.15	134.20	17	12

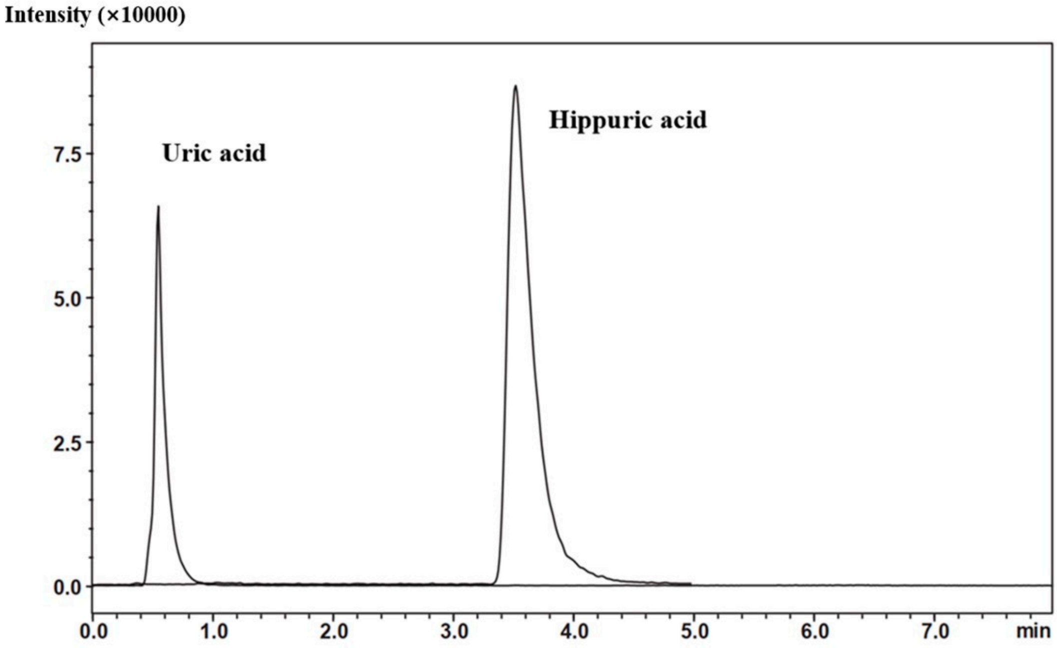

Figure S4. Base peak intensity chromatograms of uric acid and hippuric acid
